# Bcl-xL Is Required by Primary Hippocampal Neurons during Development to Support Local Energy Metabolism at Neurites

**DOI:** 10.3390/biology10080772

**Published:** 2021-08-13

**Authors:** Joseph Jansen, Madison Scott, Emma Amjad, Allison Stumpf, Kimberly H. Lackey, Kim A. Caldwell, Han-A Park

**Affiliations:** 1 Department of Human Nutrition and Hospitality Management, College of Human Environmental Sciences, The University of Alabama, Tuscaloosa, AL 35487, USA; jpjansen@crimson.ua.edu (J.J.); mlscott10@crimson.ua.edu (M.S.); ecamjad@crimson.ua.edu (E.A.); aastumpf@ua.edu (A.S.); 2 Department of Biological Sciences, College of Arts and Sciences, The University of Alabama, Tuscaloosa, AL 35487, USA; lacke003@ua.edu (K.H.L.); kcaldwel@ua.edu (K.A.C.)

**Keywords:** Bcl-xL, mitochondria, motility, ATP, neurite

## Abstract

**Simple Summary:**

B-cell lymphoma-extra large (Bcl-xL) is an anti-apoptotic protein that regulates energy metabolism in neurons. In this study, we found that primary hippocampal neurons transduced with Bcl-xL shRNA or treated with a pharmacological inhibitor of Bxl-xL had a decrease in the population of motile mitochondria. Primary hippocampal neurons lacking Bcl-xL failed to retain ATP at their neurites, which hindered the formation of complex neurite arbors, and ultimately had enhanced vulnerability to excitotoxic challenge.

**Abstract:**

B-cell lymphoma-extra large (Bcl-xL) is a mitochondrial protein known to inhibit mitochondria-dependent intrinsic apoptotic pathways. An increasing number of studies have demonstrated that Bcl-xL is critical in regulating neuronal energy metabolism and has a protective role in pathologies associated with an energy deficit. However, it is less known how Bcl-xL regulates physiological processes of the brain. In this study, we hypothesize that Bcl-xL is required for neurite branching and maturation during neuronal development by improving local energy metabolism. We found that the absence of Bcl-xL in rat primary hippocampal neurons resulted in mitochondrial dysfunction. Specifically, the ATP/ADP ratio was significantly decreased in the neurites of Bcl-xL depleted neurons. We further found that neurons transduced with Bcl-xL shRNA or neurons treated with ABT-263, a pharmacological inhibitor of Bcl-xL, showed impaired mitochondrial motility. Neurons lacking Bcl-xL had significantly decreased anterograde and retrograde movement of mitochondria and an increased stationary mitochondrial population when Bcl-xL was depleted by either means. These mitochondrial defects, including loss of ATP, impaired normal neurite development. Neurons lacking Bcl-xL showed significantly decreased neurite arborization, growth and complexity. Bcl-xL depleted neurons also showed impaired synapse formation. These neurons showed increased intracellular calcium concentration and were more susceptible to excitotoxic challenge. Bcl-xL may support positioning of mitochondria at metabolically demanding regions of neurites like branching points. Our findings suggest a role for Bcl-xL in physiological regulation of neuronal growth and development.

## 1. Introduction

B-cell lymphoma-extra large (Bcl-xL) is an anti-apoptotic member of the Bcl2 protein family [1]. Bcl-xL prevents oligomerization of pro-apoptotic Bcl2 family members such as Bax and Bak and loss of mitochondrial membrane integrity, thus inhibiting apoptotic cell death [2,3,4]. Numerous studies have demonstrated the neuroprotective functions of Bcl-xL against excitotoxicity, oxidative stress, and neurodegeneration [5,6,7,8]. In addition to its role as an anti-apoptotic protein, Bcl-xL is critical in mitochondrial function regulating neuronal bioenergetics and synaptic plasticity in the brain [9,10]. Bcl-xL closes a mitochondrial inner membrane leak channel, thereby enhancing neuronal energy metabolism with greater ATP production without requiring excess oxygen uptake [11,12,13]. Bcl-xL works upstream of dynamin-related protein 1 (Drp1) governing the number, size, and activity of the synapse [14]. Furthermore, the Bcl-xL-Drp1 complex promotes clathrin-mediated endocytosis and enhances the synaptic vesicle pool [15].

Mitochondria are dynamic organelles that change location, population, and morphology, and alterations of mitochondrial dynamics contribute to the loss of neuronal function [16,17,18,19]. Since neurons are polarized cells with each sub-cellular region possessing its own distinct energy needs [20], efficient mitochondrial trafficking is imperative to supply ATP at the metabolically active sites of neurons. In mature neurons, 20–30% of mitochondria are motile with changes in direction, speed, and momentary stationary periods, which allows for better distribution of mitochondria during the mobilization of synaptic vesicles, synaptic development, and generation of action potentials [21,22,23,24,25,26,27]. Similarly, long-range trafficking is essential to deliver damaged mitochondria back to the soma for recycling and is needed to shuttle new mitochondria throughout the neurite to replace these damaged organelles [28]. Therefore, proper mitochondrial movement is necessary for neuron functioning, and impaired mitochondrial trafficking is thought to contribute to the pathogenesis of many neurodegenerative diseases, including Alzheimer’s and Parkinson’s diseases [19,29,30]. Notably, an increasing number of studies have demonstrated that Bcl-xL has a potential role in mitochondrial dynamics [14,15,31]. Bcl-xL promotes recruitment of mitochondria at the metabolically active sites of neurons such as presynaptic sites, and it regulates mitochondrial fission and fusion, which maintains mitochondrial populations. Bcl-xL is reported to be necessary for neurite outgrowth and synapse formation [14,32]. Altogether, this evidence suggests that Bcl-xL may be pivotal in controlling local energy supply during high energy demand [14,15,31]. 

In this study, we investigated whether Bcl-xL is required during the development of primary hippocampal neurons. Neurons lacking Bcl-xL demonstrated impaired mitochondrial movement in both the anterograde and retrograde directions and were associated with local energy depletion at neurites. Failure of ATP retention at neurites impaired neurite arborization and increased susceptibility to excitotoxic challenges. We suggest that Bcl-xL is an important contributor to the physiologic development of neurite complexity by supporting neurite energy retention.

## 2. Materials and Methods

### 2.1. Culture of Primary Hippocampal Neurons

Primary rat hippocampal neurons were prepared from rat feti (Sprague-Dawley, day 18 of gestation; Envigo, Indianapolis, IN, USA) as described previously [5,32,33,34]. Briefly, neurons (0.3 × 10^6^ cells/35 mm plate) were seeded on poly-l-lysin coated plates and grown in a neurobasal medium supplemented with B-27, glutamine, and antibiotics (Thermo Fisher Scientific, Waltham, MA, USA) for 21 days in vitro (DIV). shRNA lentiviral particle transduction: Primary hippocampal neurons were transduced with control shRNA lentiviral particles or Bcl-xL shRNA lentiviral particles (Santa Cruz Biotechnology, Dallas, TX, USA); copGFP control lentiviral particles or Bcl-xL shRNA-GFP lentiviral particles (Santa Cruz Biotechnology) at DIV 7. *Glutamate treatment:* Glutamate (Sigma-Aldrich, St. Louis, MO, USA) was freshly prepared in sterile PBS (Thermo Fisher Scientific) and added to the cell culture medium (final concentration: 20 μM). The vehicle control group for the glutamate experiment was treated with isovolumetric sterile PBS. ABT-263 treatment: ABT-263 was prepared in dimethyl sulfoxide (DMSO) and added to the cell culture medium (final concentration 1 μM). The vehicle control group for ABT-263 was treated with isovolumetric DMSO. The protocol was approved by the Institutional Animal Care Committee (IACUC) of the University of Alabama, Tuscaloosa, AL, USA.

### 2.2. Live Imaging and Mitochondrial Motility Analysis

Mitochondria were labeled with CellLight^TM^ mitochondria-RFP (Thermo Fisher Scientific, Waltham, MA, USA) and image sequences were generated via fluorescent imaging with images taken every 60 s over a 60 min period. Image sequences were then used to generate kymographs using the KymoAnalyzer 1.01 software for ImageJ [35]. To generate the kymographs, image sequences were opened using the Batch Kymograph Generation plugin within KymoAnalyzer. Each neurite was then traced from the soma to the distal tip, thereby giving the track directionality in KymoAnalyzer. Anterograde movement is defined as a movement towards the distal tip, while retrograde movement is a movement towards the soma. Separate kymograph images were generated for each neurite showing the movement within the neurite throughout the image sequence. Next, kymograph images were opened using the Tracks plugin within KymoAnalyzer, and tracks were traced from the top of the image to the bottom of the image. Traces were completed for each kymograph individually and saved for analysis. The CargoPopulation, NetCargoPopulation, Segments, and PoolData plugins within KymoAnalyzer were then run successively on the folder of saved traces, and data was generated and exported. Each data metric is defined below: % anterograde track: The percentage of total tracks that showed only anterograde movement; any track that reversed directions are not included; % net anterograde track: The percentage of total tracks that showed net anterograde movement; includes tracks that reversed direction so long as the net movement was in the anterograde direction; % retrograde track: The percentage of total tracks that showed only retrograde movement; any tracks that reversed directions are not included; % net retrograde track: The percentage of total tracks that showed net retrograde movement; includes tracks that reversed direction so long as the net movement was in the retrograde direction; % stationary track: The percentage of total tracks that showed no movement; % net stationary track: The percentage of total tracks that showed no net movement; tracks that moved, but returned to their original location are included; Anterograde density: The number of tracks showing anterograde movement per micrometer of neurite (tracks/µm); Retrograde density: The number of tracks showing retrograde movement per micrometer of neurite (tracks/µm); Reversal density: The number of tracks that reversed direction at least once per micrometer of neurite (tracks/µm); Stationary density: The number of tracks showing no movement per micrometer of neurite (tracks/µm).

### 2.3. Analysis of Neurite Branching

Primary hippocampal neurons transduced with GFP control lentiviral particles (copGFP, Santa Cruz Biotechnology) or Bcl-xL shRNA-GFP lentiviral particles (Santa Cruz Biotechnology) were imaged with a Zeiss Axio Vert.A1 microscope (Zeiss, Oberkochen, Germany). Sholl analysis was used to quantify neurite branches as previously described [36,37]. In brief, fluorescent micrographs of primary hippocampal neurons were opened using the Simple Neurite Tracer plugin for ImageJ (National Institutes of Health, Bethesda, MD, USA), and neurites from the soma and daughter branches from the neurites were selected using the Path Manager function. Saved traces were analyzed using Sholl analysis, and a Sholl profile was created with the number of intersections at the specific radium, and micrographs were converted with pseudo-color (16-color annotation). 

### 2.4. Measurement of the ATP/ADP Ratio

Primary hippocampal neurons were transfected at DIV7 with GW1-PercevalHR, a sensor developed in Gary Yellen’s laboratory [38,39]. Neuronal images (excitation 488 nm and emission 530 nm/excitation 405 nm and emission 530 nm) were obtained using a Nikon C2 Laser Scanning Confocal Microscope (Nikon, Tokyo, Japan) at the Optical Analysis Facility at the University of Alabama. The ratio of fluorescence intensities was calculated as F_488nm_/F_405nm_. Pseudo-color ratiometric images were made using NIS-Elements 5.11 with the Ratio View Live Ratio Graphing Module (Nikon) [40].

### 2.5. Measurement of Mitochondrial Potential (Δψ)

Mitochondrial membrane potential (Δψ) was measured using the fluorescent lipophilic cationic dye tetramethylrhodamine methyl ester (TMRM) (Thermo Fisher Scientific, Waltham, MA, USA), which accumulates within mitochondria in a potential-dependent manner [41,42]. Primary neurons were stained with TMRM (5 nM in DMSO) for 30 min at 37 °C in the dark. Images were taken with a Zeiss Axiovert A1 microscope using a consistent exposure time and TMRM fluorescence density was analyzed using AxioVision 4.9.

### 2.6. Immunoblots

Neurons were scraped and lysed in 1X cell signaling buffer (Cell Signaling Technology, Danvers, MA, USA) and protein concentration was determined using BCA protein reagents (Thermo Fisher Scientific, Waltham, MA, USA). Samples (50–100 µg of protein/lane) were separated on a 4–12% SDS-polyacrylamide gel (Bio-Rad, Hercules, CA, USA) and probed with anti-Bcl-xL antibody (1:1000, Cell Signaling Technology, Danvers, MA, USA) and anti-beta actin (Sigma-Aldrich, 1:1000). Scanned images were analyzed using ImageJ software (National Institutes of Health, Bethesda, MD, USA). 

### 2.7. Calcein-AM, and Propidium Iodide (PI) Staining

Viable or dead cells were stained with Calcein-AM or PI as previously described [5]. After treating neurons with glutamate (20 μM in sterile PBS), Calcein-AM (25 nM in DMSO), PI (0.5 μM in sterile PBS), or Hoechst (1 μg/mL in sterile PBS) (Thermo Fisher Scientific, Waltham, MA, USA) was added into the culture medium for 30 min at 37 °C in the dark. Micrographs were taken using a Zeiss Axiovert A1 microscope (Zeiss, Oberkochen, Germany) using a consistent exposure time. The number of PI-positive neurons, Hoechst positive neurons, or calcein fluorescence density per cell was analyzed using AxioVision 4.9. 

### 2.8. Fluo-4 Staining

Primary hippocampal neurons were treated with 2.5 µM Fluo-4 (Invitrogen) solution prepared in a light-protected vessel, then incubated for 30 min at 37 °C as per the manufacturer’s protocol [43]. Micrographs were taken using a Zeiss Axiovert A1 microscope (Zeiss, Oberkochen, Germany). Fluo-4 fluorescence density per cell was analyzed using AxioVision 4.9. 

### 2.9. Immunocytochemistry

Primary hippocampal neurons fixed in 10% buffered formalin were blocked in 10% goat serum for 1 h, then incubated with anti-Bassoon (1:10, Santa Cruz Biotechnology, Dallas, TX, USA), anti-synaptophysin (1:10, Santa Cruz Biotechnology, Dallas, TX, USA), anti-MAP2 (1:100, Cell Signaling Technology, Danvers, MA, USA) antibodies overnight at 4 °C. Cells were washed and incubated with Alexa-fluor 488 antibody or Alexa-568 antibody (1:200 dilution; Invitrogen, Molecular Probes, Carlsbad, CA, USA) for 1 h at room temperature and mounted on glass slides. Images were taken with a Zeiss Axiovert A1 microscope and processed using AxioVision 4.9.

### 2.10. Statistical Analysis

Data are reported as the mean ± SEM of at least three independent cultures with multiple independent experimental designs, such as independent neuronal isolation, independent performance dates, and independent plates within a culture using separately prepared reagents. All quantitative graphs were made from at least three independent neuronal isolations. *p* < 0.05 was considered statistically significant. *p* values are provided in figure legends.

## 3. Results

### 3.1. Bcl-xL Depletion Lowers ATP/ADP Ratios at Neurites of Primary Hippocampal Neurons

**Figure 1:**
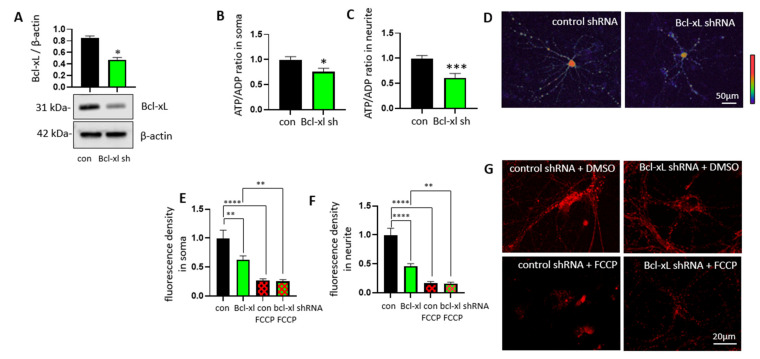
Bcl-xL depletion alters ATP/ADP ratio in primary hippocampal neurons. Primary rat hippocampal neurons were transduced with control shRNA or Bcl-xL shRNA. Immunoblotting (**A**) was performed to show depletion of the Bcl-xL protein levels in Bcl-xL shRNA transduced neurons (*n* = 4, * *p* = 0.0286, Mann–Whitney test). ATP/ADP ratio (**B**,**C**) was measured using the GW1-PercevalHR sensor (F_488nm_/F_405nm_). Bcl-xL depleted neurons showed lower ATP/ADP ratios in both the soma (*n* = 20) and neurites (*n* = 20). * *p* < 0.05, and *** *p* < 0.001, two-tailed Student’s *t*-test. Scale bar = 50 μm. Pseudocolour ratiometric micrographs (**D**) of neurons indicating the ATP/ADP ratio using the PercevalHR sensor. Red, higher ATP/ADP; Blue, lower ATP/ADP. Primary hippocampal neurons lacking Bcl-xL showed a significantly decreased TMRM signal (**E**–**G**), indicating a loss of mitochondrial inner membrane potential (Δψ) (*n* = 12). ** *p* < 0.01, and **** *p* < 0.0001, two-tailed Student’s *t*-test. Scale bar = 20 μm.

Bcl-xL is reported to improve neuronal energy metabolism by enhancing mitochondrial ATP production via direct interaction with the F_1_Fo ATP synthase [12,13]. However, it is still unclear if Bcl-xL can control energy metabolism at a specific region in neurons. In order to investigate the roles of Bcl-xL on local ATP levels, primary hippocampal neurons isolated from E18 rats were transduced with either Bcl-xL shRNA or control shRNA at days in vitro 7 (DIV7) (Figure 1A; Supplementary Figure S1). We have previously shown that transduction of Bcl-xL shRNA takes about 2 weeks to deplete Bcl-xL in primary hippocampal neurons [32], thus neurons were incubated for additional 2 weeks. Additionally, we applied a GW1-PercevalHR probe to monitor ATP/ADP ratio in primary hippocampal neurons [38]. The probe undergoes a conformational change upon binding to ATP, resulting in changes in the fluorescence signal. The ATP/ADP ratio in live neurons is further converted to a pseudocolor ratiometric image to visualize ATP levels at specific regions of neurons such as the soma and neurites (Figure 1B–D). Although transduction with Bcl-xL shRNA lowered ATP/ADP ratio throughout whole neurons, including soma and neurites, we found that ATP levels at the neurite were greatly impacted by Bcl-xL depletion. We further applied tetramethylrhodamine (TMRM) to measure mitochondrial inner membrane potential (Δψ). Primary hippocampal neurons lacking Bcl-xL significantly decreased TMRM positive signals at their neurites, indicating loss of mitochondrial function and impaired energy metabolism (Figure 1E–G). TMRM fluorescence was significantly decreased after treatment with FCCP, an uncoupler of oxidative phosphorylation.

### 3.2. Bcl-xL Depletion Impairs Normal Mitochondrial Motility Patterns in Neurites of Primary Hippocampal Neurons

**Figure 2:**
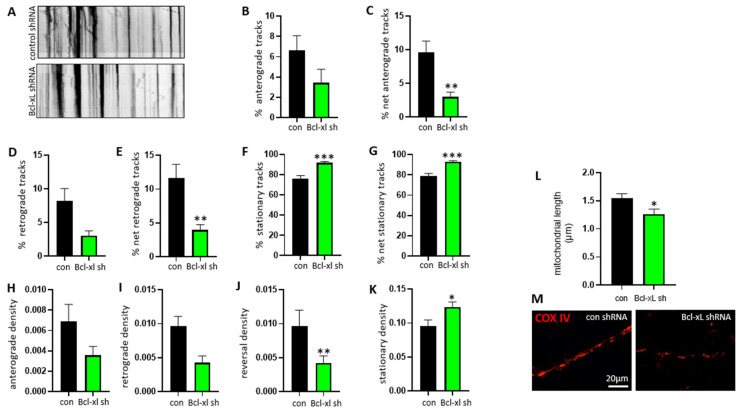
Bcl-xL depletion disrupts mitochondrial motility in primary hippocampal neurons. Primary rat hippocampal neurons were transduced with control shRNA or Bcl-xL shRNA. Kymographs (**A**) were produced and analysis using the KymoAnalyzer was completed to give the percentage of mitochondria that showed mitochondria moving in the following directions: only anterograde (**B**), *n* = 40, net anterograde (**C**), *n* = 40, ** *p* = 0.0022, Mann-Whitney test, only retrograde (**D**), *n* = 40, net retrograde (**E**), *n* = 40, ** *p* = 0.0075, Mann–Whitney test, stationary (**F**), *n* = 40, *** *p* = 0.0001, Mann–Whitney test, and net stationary (**G**), *n* = 40, *** *p* = 0.0002, Mann–Whitney test. The analysis also provided the mitochondrial density (number of mitochondria/µm) for mitochondria exhibiting anterograde (**H**), *n* = 40 or retrograde (**I**), *n* = 40 motion, as well as mitochondria that reversed directions (**J**), *n* = 40, ** *p* = 0.0029, Mann–Whitney test during the image sequence and stationary mitochondria (**K**), *n* = 40, * *p* = 0.0177, Mann–Whitney test. The COX IV protein was labeled to visualize mitochondria (**L**,**M**), and mitochondria length was measured (*n* = 63). * *p* < 0.05, two-tailed Student’s *t*-test. Scale bar = 20 μm.

To determine whether Bcl-xL is required to localize mitochondria at neurites, we monitored mitochondrial motility using neurons with or without expressing Bcl-xL. Primary hippocampal neurons were transduced with control shRNA or Bcl-xL shRNA followed by mitochondrial labeling using mito-RFP BacMam2.0. Image sequences visualizing mitochondrial movement were converted to kymographs (Figure 2A), and motility parameters including anterograde and retrograde movement were applied. Bcl-xL-depleted neurons demonstrated abnormal mitochondrial movement throughout the neurite compared to control neurons. The percentage of mitochondria exhibiting only anterograde movement was reduced in Bcl-xL depleted neurons, but the difference was not significant (Figure 2B). However, when including mitochondria that reversed direction during the imaging period, the decrease in the percentage of mitochondria with net anterograde movement was significant (Figure 2C). The percentage of mitochondria showing only retrograde movement was lower in Bcl-xL depleted neurons (Figure 2D); moreover, there was also a significant decrease in the overall net retrograde movement in Bcl-xL depleted neurons (Figure 2E). In addition, the percentage of stationary (Figure 2F) and net stationary (Figure 2G) mitochondria were significantly higher in Bcl-xL depleted neurons. 

Bcl-xL depletion affected the amount of mitochondrial movement. We, therefore, tested if Bcl-xL depletion affected the density of mitochondria in the neurite. Primary hippocampal neurons expressing Bcl-xL also displayed higher densities of moving mitochondria and number of motile mitochondria per micrometer of neurite than Bcl-xL depleted neurons (Figure 2H–J). The density of stationary mitochondria (Figure 2K) was significantly higher in Bcl-xL depleted neurons than in control neurons. Furthermore, the COX IV protein was labeled to visualize mitochondria. Neurons were imaged and mitochondria length was measured (Figure 2L,M). It was found that the Bcl-xL depleted neurons had decreased mitochondria length when compared to control neurons. 

### 3.3. Treatment with ABT-263 Impairs Mitochondrial Motility in Primary Hippocampal Neurons

**Figure 3:**
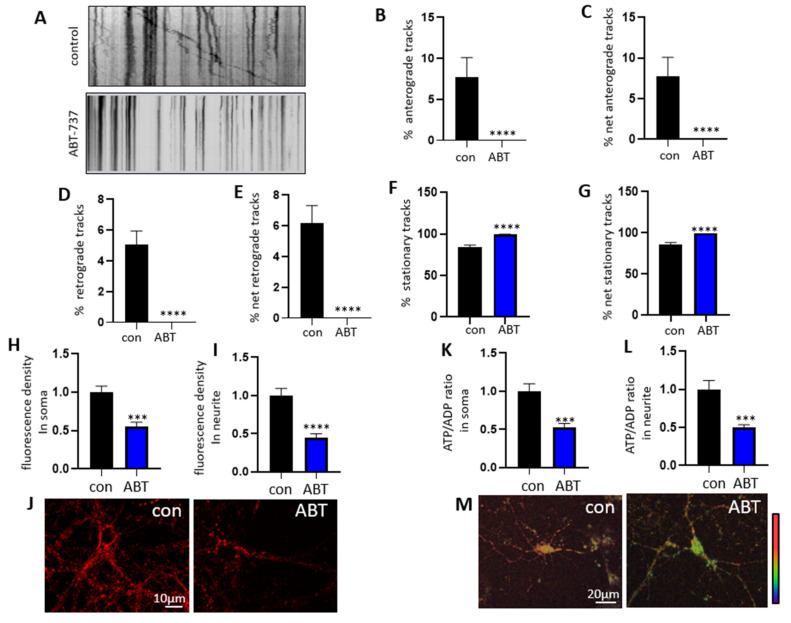
Treatment with ABT-263 impairs mitochondrial motility in primary hippocampal neurons. Primary hippocampal neurons were treated with or without ABT-263 (1 μM). Neurons treated with ABT-263 were analyzed using the KymoAnalyzer (**A**) and the following metrics were generated: % anterograde (**B**), *n* = 57, **** *p* < 0.0001, Mann–Whitney test, % net anterograde (**C**), *n* = 57, **** *p* < 0.0001, Mann–Whitney test, % retrograde (**D**), *n* = 48, **** *p* < 0.0001, Mann-Whitney test, % net retrograde (**E**), *n* = 48, **** *p* < 0.0001, Mann–Whitney test, % stationary (**F**), *n* = 48, **** *p* < 0.0001, Mann–Whitney test, and % net stationary tracks (**G**), *n* = 48, **** *p* < 0.0001, Mann–Whitney test. Neurons were treated with TMRM and fluorescence density was measured (**H**–**J**), *n* = 12. *** *p* < 0.001, and **** *p* < 0.0001, two-tailed Student’s *t*-test. Scale bar = 10 µm. (**K**–**M**), The PercevalHR Sensor (F_488nm_/F_405nm_) was applied and psuedocolour ratiometric microgaphs were used to indicate the ATP/ADP ratio (*n* = 12). *** *p* < 0.001, two-tailed Student’s *t*-test. Red, higher ATP/ADP; Blue, lower ATP/ADP. Scale bar = 20 µm.

Furthermore, we applied a pharmacological approach to target Bcl-xL Primary hippocampal neurons were treated with or without 1 µM ABT-263, an inhibitor of Bcl-xL. As previously described, kymographs were generated (Figure 3A) and the analysis was performed using the KymoAnalyzer. Neurons treated with ABT-263 showed significantly decreased retrograde and anterograde movement (Figure 3B–E), whereas the percentage of stationary mitochondria was significantly increased compared to the control neurons (Figure 3F,G). 

TMRM fluorescence was also analyzed in ABT-263-treated neurons to measure mitochondrial inner membrane potential (Δψ) (Figure 3H–J). ABT-263-treated neurons showed a significantly decreased TMRM positive signal in both the soma and the neurite. The GW1-PercevalHR sensor was applied to measure ATP/ADP ratio in primary hippocampal neurons. It was found that ABT-263-treated neurons had decreased ATP/ADP ratio in both the neurite and soma, indicating impaired energy retention (Figure 3K–M).

### 3.4. Bcl-xL Depletion Impairs Neurite Arborization and Synapse Formation in Primary Hippocampal Neurons

**Figure 4:**
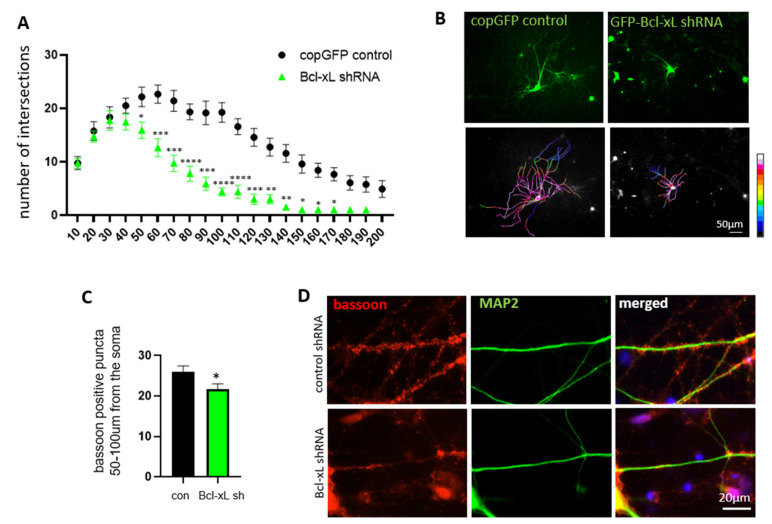
Bcl-xL depletion impairs neurite arborization and synapse formation in primary hippocampal neurons. Primary hippocampal neurons were transduced with conGFP control or Bcl-xL shRNA-GFP. The Sholl analysis (**A**) determined the number of neurite intersections (*n* = 12). Pseudocolour images (**B**) show a qualitative difference in neurite intersections resulting from Bcl-xL depletion. Red, higher intersections; blue, fewer intersections. Scale bar = 50 µm. Immunocytochemistry (**C**) was performed, and Bassoon-positive puncta were counted from 50–100 µm from the soma (*n* = 63). * *p* < 0.05, ** *p* < 0.01, *** *p* < 0.001, and **** *p* < 0.0001, two-tailed Student’s *t*-test. (**D**) Red, Bassoon; Green, MAP2; Blue, DAPI. Scale bar = 20 μm.

We have previously shown that Bcl-xL is necessary for neurite outgrowth during the development of primary hippocampal neurons [32]. To test if Bcl-xL-dependent ATP retention at neurites is required to maintain normal neurite morphology, we measured the number of neurite branches by applying the Sholl analysis. Primary hippocampal neurons transduced with either control-GFP or Bcl-xL shRNA-GFP were analyzed by tracing the neurite projections from the soma. The number of intersections, or branch points, was totaled at the radius from the soma in 10 µm increments. Bcl-xL depleted neurons and control neurons had similar amounts of intersections up to 40 µm from the soma. At 50 µm and beyond, however, Bcl-xL depleted neurons had significantly decreased levels of intersections (Figure 4A). Furthermore, no Bcl-xL depleted neuron showed any branching past 190 µm from the soma, while control neurons showed branching up to 200 µm from the soma, suggesting again that Bcl-xL plays a large role in neurite growth and development. Original fluorescent micrographs were converted to pseudocolor with 16-color annotation: red indicates more intersections and blue indicates fewer intersections. Pseudocolor images visualized primary hippocampal neurons lacking Bcl-xL showed decreased neurite branches suggesting the importance of Bcl-xL in the formation of neurite complexity (Figure 4B). 

We then further tested to see if Bcl-xL depletion impacted synapse formation. Immunocytochemistry was performed on primary hippocampal neurons (Figure 4C,D) to visualize Bassoon, a scaffolding protein that is important to the structure and function of the pre-synapse [44]. Cells were imaged and Bassoon-positive puncta were counted from 50–100 µm from the soma. This range was chosen because at 50 µm from the soma neurite arborization begins to differ between control and Bcl-xL depleted neurons, and at any distance further than 100 µm from the soma Bcl-xL depleted neurons show very little neurite branching and are underdeveloped compared to control neurites at the same distance. Thus, markers of the pre-synapse cannot fully be attributed to Bcl-xL protein depletion at distances further than 100 µm. We found that Bassoon-positive puncta were significantly decreased in Bcl-xL depleted neurons compared to the control neurons. This data suggests that Bcl-xL depletion impairs proper neurite growth, branching, and synapse formation (Figure 4C,D; Supplementary Figure S2).

### 3.5. Bcl-xL Depletion Increases the Susceptibility of Primary Hippocampal Neuron to Excitotoxicity

**Figure 5:**
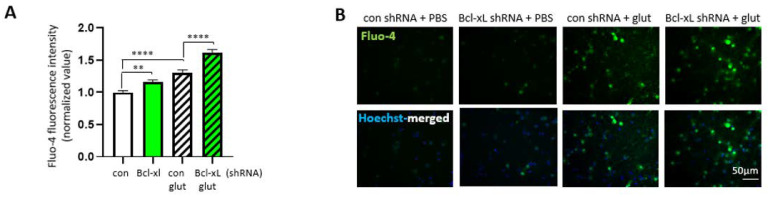

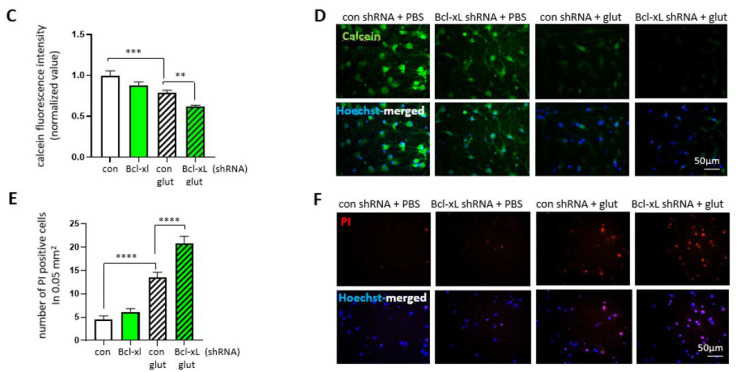
Bcl-xL depletion makes primary hippocampal neurons more susceptible to excitotoxicity. Primary hippocampal neurons transduced with control shRNA or Bcl-xL shRNA were treated with or without glutamate to induce excitotoxicity. Neurons were treated with Fluo-4 (**A**,**B**) to measure intracellular calcium concentration (*n* = 121). ** *p* < 0.01 and **** *p* < 0.0001, Kruskal–Wallis test with Dunn’s multiple comparisons test. Calcein-AM and Hoechst staining (**C**,**D**) shows the proportion of healthy cells via fluorescent image analysis (*n* = 100). ** *p* < 0.01, and *** *p* < 0.001, one-way ANOVA with a Tukey’s post-hoc-analysis. Scale bar = 20 μm. PI and Hoechst staining (**E**,**F**) shows the proportion of dead cells via fluorescent image analysis (*n* = 20). **** *p* < 0.0001, one-way ANOVA with a Tukey’s post-hoc analysis. Scale bar = 50 μm.

Alteration of neurite morphology does not cause the immediate death of developing neurons; however, it does increase the vulnerability of neurons during maturation [32]. To determine if impaired neurite branching in Bcl-xL depleted neurons is associated with susceptibility of neurons against neurotoxic insult, we analyzed the viability of neurons under treatment with the excitotoxic neurotransmitter, glutamate. After transduction with control shRNA or Bcl-xL shRNA, primary hippocampal neurons were treated with or without glutamate or solvent control for 24 h. Neurons were treated with Fluo-4 to measure intracellular calcium concentration (Figure 5A,B). Bcl-xL depleted neurons showed increased Fluo-4 signal compared to control neurons without treatment with glutamate. Furthermore, Bcl-xL depleted neurons treated with glutamate showed a greatly increased Fluo-4 positive signal compared to control neurons treated with glutamate. These data suggest that treatment with glutamate greatly increases intracellular calcium and that Bcl-xL depleted neurons are much more susceptible to this increase in calcium concentration. 

Neurons were then labeled with calcein-AM, to indicate live, healthy cells, and Hoechst, to show all cells present. Consistent with our previous report [45], treatment with glutamate significantly lowers the calcein-positive fluorescent signal. Interestingly, glutamate-induced excitotoxicity was exacerbated in Bcl-xL depleted neurons (Figure 5C,D). Similarly, neurons lacking Bcl-xL showed significantly increased propidium iodine (PI) positive cells under glutamate challenge, indicating increased levels of apoptotic and necrotic death (Figure 5E,F). These results suggest that Bcl-xL plays a role in protection against excitotoxicity in neurons.

## 4. Discussion

Bcl-xL is traditionally known as a blocker of apoptotic death via its ability to bind pro-apoptotic proteins. In this study, we found an additional function of Bcl-xL under physiological conditions. Depletion of Bcl-xL significantly decreased the population of motile mitochondria in primary hippocampal neurons, and this may hinder the delivery of mitochondria, impairing local energy metabolism at neurites. Indeed, we found that neurons transduced with Bcl-xL shRNA failed to maintain mitochondrial inner membrane potential and depleted ATP at their neurites, arresting arborization of neurites compared to neurons expressing Bcl-xL. Although Bcl-xL depletion did not immediately change neuronal viability, failure to achieve neurite complexity eventually increased the vulnerability of neurons against excitotoxic challenge.

Bcl-xL regulates the efficiency of ATP production in mitochondria via direct interaction with the F_1_Fo ATP synthase [12,13]. In particular, Bcl-xL binds directly to the β-subunit of the F1 complex, the key subunit that phosphorylates ADP to produce ATP [12]. Interaction between Bcl-xL and the F_1_Fo ATP synthase enhances the efficiency of ATP production by blocking a leak channel activity. Although there are several candidates to form mitochondrial permeability transition pore (mPTP), the c-subunit of the F_1_Fo ATP synthase, a ring-shaped subunit found in the Fo complex, exhibits non-selective voltage-gated and calcium-activated channel activities resembling mPTP [46,47,48]. A recent study demonstrated that the monomeric form of the F_1_Fo ATP synthase showed mPTP-like activities, whereas treatment with oligomycin A closed the channel [47]. Since motor proteins such as dynein and kinesin contain ATP hydrolyzing sites [49,50,51], inefficient operation of the F_1_F_o_ ATP synthase in neurons lacking Bcl-xL may lead to failure of intracellular transport. Additionally, we found that primary hippocampal neurons expressing Bcl-xL have approximately 22% motile and 78% stationary mitochondrial populations, whereas neurons lacking Bcl-xL maintained only 7% motile mitochondria (Figure 2F,G). Consistently, neurons treated with ABT-263 completely lost mitochondrial motility (Figure 3A,B). Since we currently achieve an 80–90% transfection rate with Bcl-xL shRNA [32], 7% motile mitochondria may represent an un-transfected population in the Bcl-xL shRNA group. ABT-263 is a BH3 mimetic that blocks the interaction between Bcl-xL and other pro-apoptotic BH3 proteins. The BH3 only protein Bim is known to bind to Bcl-xL [52,53], and it is previously reported to form a complex with dynein, a motor protein that carries mitochondria [54,55]. Therefore, it may be important to further test if Bcl-xL-dependent mitochondrial motility is due to the direct interaction between Bcl-xL and the motor complex or regulation of the efficiency of the F_1_Fo ATP synthase.

Previously, we reported that depletion of Bcl-xL does not directly cause neuronal death under physiological conditions, but neurons lacking Bcl-xL show significantly altered neurite morphology leading to death during hypoxic insult [32]. Similarly, in this study, we found that neurons lacking Bcl-xL arrest the formation of neurite branches, and the failure to achieve neurite complexity, are associated with the susceptibility of neurons against excitotoxicity. Notably, neurites proximal to the soma (<40 μm) developed normally in Bcl-xL shRNA transduced neurons, whereas impaired arborization severely occurred in distal regions of neurites. We have demonstrated that transduction of Bcl-xL shRNA takes about 2 weeks to deplete Bcl-xL in primary hippocampal neurons [32]. Thus, neurons may be able to maintain adequate ATP during early development. However, when neurons start their maturity, grown in vitro for approximately 2 weeks, Bcl-xL depleted neurites failed to form daughter branches.

In this study, we showed that full-length Bcl-xL is required for neurite development. Bcl-xL supports local energy retention at neurites and enhances both the efficiency of ATP production and the recruitment of mitochondria to intracellular locations. We suggest a physiological function of Bcl-xL during neuronal development and under neurotoxic challenge.

## Data Availability

The data presented in this study are available on request from the corresponding author.

## Supplementary Materials

The following are available online at https://www.mdpi.com/article/10.3390/biology10080772/s1, Figure S1: Full Western Blot; Figure S2: Immunocytochemistry.
