# Supplementary material for: Bcl-xL Is Required by Primary Hippocampal Neurons during Development to Support Local Energy Metabolism at Neurites
Source: Biology (Basel). 2021 Aug 13;10(8):772. doi: 10.3390/biology10080772 (PMC8389656; doi:10.3390/biology10080772)
Supplement: Supplementary file 1 [file biology-10-00772-s001.zip › biology-1334897-supplementary.pdf]

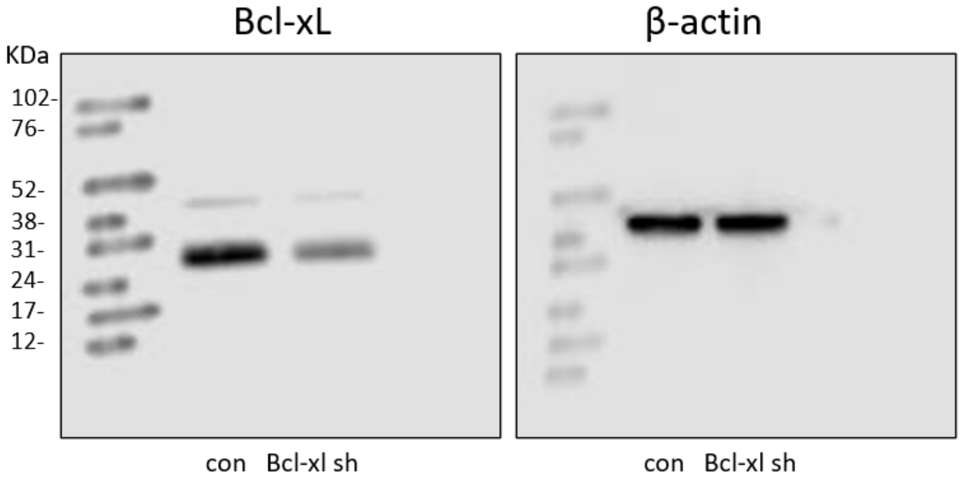

Supplement S1. Primary hippocampal neurons transduced with Bcl-xL shRNA decreased protein levels of Bcl-xL. Primary rat hippocampal neurons were transduced with control shRNA or Bcl-xL shRNA. Depletion of Bcl-xL protein levels in Bcl-xL shRNA transduced neurons was shown via immunoblotting.

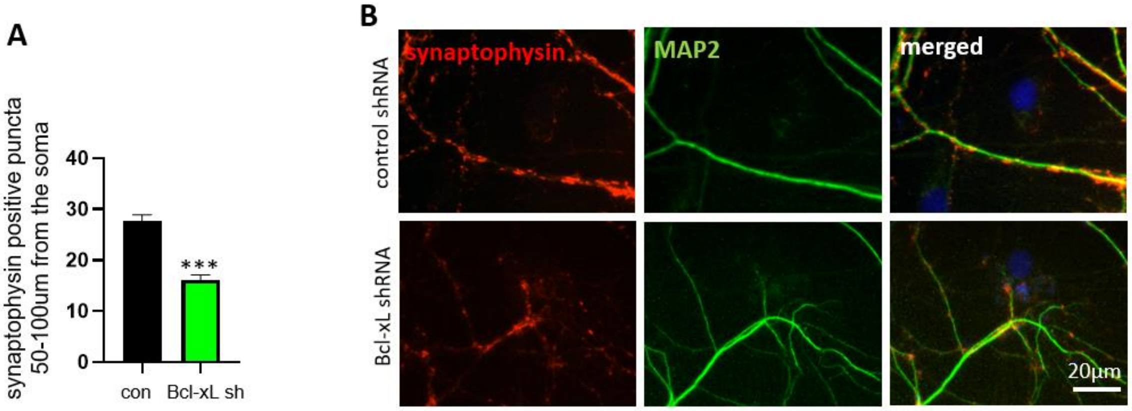

Supplement S2. Bcl-xL depletion decreases synaptophysin positive puncta. Immunocytochemistry (**A-B**) was performed and synaptophysin positive puncta were counted from 50-100µm from the soma. Bcl-xL depleted neurons show a significant decrease in synaptophysin positive puncta (n=42). ****P*< 0.001, two-tailed Student's *t*-test. Red, synaptophysin; Green, MAP2, Blue, DAPI, Scale bar=20µm.
